# BABY-LED WEANING, AN OVERVIEW OF THE NEW APPROACH TO FOOD INTRODUCTION: INTEGRATIVE LITERATURE REVIEW

**DOI:** 10.1590/1984-0462/2020/38/2018084

**Published:** 2020-01-13

**Authors:** Melisa Sofia Gomez, Ana Paula Toneto Novaes, Janaina Paulino da Silva, Luciane Miranda Guerra, Rosana de Fátima Possobon

**Affiliations:** a Universidade Estadual de Campinas, Piracicaba, SP, Brazil.

**Keywords:** Infant nutrition, weaning, Feeding behavior, Feeding methods, Child health, Nutrição do lactente, Desmame, Comportamento alimentar, Métodos de alimentação, Saúde da criança

## Abstract

**Objective:**

To analyze the scientific literature on Baby-Led Weaning with an integrative literature review to identify risks and benefits.

**Data source:**

The databases used were: National Library of Medicine (MEDLINE), Latin American and Caribbean Literature in Health Sciences (LILACS – *Literatura Latino-Americana e do Caribe em Ciências da Saúde*), US National Library of Medicine (PubMed), and Virtual Health Library (BVS – *Biblioteca Virtual em Saúde*) in December 2017. The inclusion criteria established were publications in English with the descriptor “baby-led weaning” in the heading, abstract, or keywords, classified as original articles, of primary nature, and available online and in full. We excluded review articles, editorials, letters to the editor, critical commentaries, and books on the subject, as well as articles not available in full and duplicates.

**Data summary:**

We identified 106 articles, of which 17 met the selection criteria. The Baby-Led Weaning method was significantly associated with the baby’s satiety, the start of complementary feeding, and adequacy of weight gain. On the other hand, choking and the intake of micronutrients were negatively associated, however with no statistical differences.

**Conclusions:**

Despite the benefits found, the risks still deserve attention and should be investigated with longitudinal randomized controlled studies to ensure the safety of the method when practiced exclusively.

## RESUMO

**Objetivo:**

Analisar a literatura científica referente ao desmame guiado pelo bebê (*Baby-Led Weaning*) por meio de revisão integrativa de literatura a fim de identificar riscos e benefícios.

**Fonte de dados:**

As bases de dados utilizadas foram: National Library of Medicine (MEDLINE), Literatura Latino-Americana e do Caribe em Ciências da Saúde (LILACS), US National Library of Medicine (PubMed) e Biblioteca Virtual em Saúde (BVS); e a busca foi realizada em dezembro de 2017. Os critérios de inclusão estabelecidos foram publicações em inglês com o descritor “*baby-led weaning*” no título, resumo ou palavras-chave em artigos classificados como originais de natureza primária, disponibilizados *online* e na íntegra. Excluíram-se artigos de revisão, editoriais, cartas ao editor, comentários críticos e livros abordando o assunto, assim como artigos não disponíveis na íntegra e duplicatas.

**Síntese dos dados:**

Identificaram-se 106 artigos, dos quais 17 faziam parte do critério de seleção. O método *Baby-Led Weaning* teve associação significativa com a saciedade do bebê, início da alimentação complementar e adequação de ganho de peso. Já o engasgo e a ingestão de micronutrientes foram associados negativamente, contudo sem diferenças estatísticas.

**Conclusões:**

Apesar dos benefícios apontados, os riscos ainda merecem atenção por meio de pesquisas longitudinais controladas e randomizadas para fornecer mais segurança para a sua prática de forma exclusiva.

## INTRODUCTION

Complementary feeding is understood as an important physiological milestone in the life of the baby, given that adequate nutrition, capable of providing sufficient nutritional quantity and quality, is essential to ensure the growth and overall development in its fullest potential.^1^

Considering the importance of complementary feeding, the World Health Organization (WHO) recommends starting it after the sixth month of the infant’s life, as all the baby’s nutritional needs are met exclusively by breastfeeding until this age.^2^

The food introduction recommended by WHO is considered traditional, starting with purees and gradually increasing the consistency until the infant reaches 12 months of age to respect the learned masticatory movements and swallowing ability.^3^ Both the Brazilian Society of Pediatrics^4^ and the Ministry of Health^5^ give the same recommendation and even encourage the family to eat together in a harmonious environment to establish healthy habits. In addition, they emphasize the need to pay attention to the baby’s satiety signals.

In contrast to the traditional model, the British nurse Gill Rapley developed a new approach to food introduction in 2008. This method is called Baby-Led Weaning (BLW), considered an alternative that encourages self-feeding after the sixth month of life. In this model, foods, preferably those consumed by the family, are offered to the infant as finger foods, allowing the child to feed alone, promoting his or her independence and an intense sensory exploration, unlike the traditional method, in which the parents spoon-feed purees to their children (parent-led) and gradually adapt the food texture.^6,7^ This method is gaining popularity among parents, particularly in the United Kingdom and New Zealand, whose departments of health recommend offering finger foods since the beginning of food introduction and after the seventh month, respectively.^8,9^

In the last decade, several scientific pieces of research and books on BLW were translated into more than 15 languages. However, despite the benefits disclosed about the method, health professionals are reluctant to advise the adoption of this new approach, given the lack of evidence of high scientific rigor, considering the many concerns raised about the model.^2^ The discussions revolve around its possible negative impact on the child’s health, due to the increased risk of choking and a greater probability of low energy intake and micronutrient consumption, especially iron, since the child determines the quantity and quality of the food by choosing among the various options presented to him or her at mealtimes.^6,10^

However, the approach offers several benefits, such as obesity prevention, as it respects self-regulation, higher consumption of fruits and vegetables, better development of motor skills, and positive effects on parent behavior. The child is encouraged to participate in family meals, with no pressure regarding time and amount of food consumed, and interact with the food, widely exploring sensory aspects, through different textures, and consequently creating a better relationship with food.^11^

Considering the need for health professionals to know the impact of BLW, this work aimed to provide an overview of the scientific evidence on the approach that has brought new concepts to food introduction.

## METHOD

This is an integrative literature review that analyzed articles on the BLW method of food introduction for six-month-old babies. The present study was designed based on the six steps recommended for the elaboration of a quality integrative review:^12,13^

- Selecting the theme and guiding question.
- Establishing inclusion and exclusion criteria.
- Sampling (selecting the articles).
- Categorizing the selected articles.
- Analyzing and interpreting data.
- Summarizing the knowledge by presenting the integrative review.

Three independent reviewers performed the initial step, which corresponded to the search of the descriptor in English: Baby-Led-Weaning. They searched the electronic databases National Library of Medicine (MEDLINE), Latin American and Caribbean Literature in Health Sciences (LILACS – *Literatura Latino-Americana e do Caribe em Ciências da Saúde*), US National Library of Medicine (PubMed), and Virtual Health Library (BVS – *Biblioteca Virtual em Saúde*) in English in December 2017.

The inclusion criteria established were: publications on BLW, classified as original articles of primary nature, available online and in full; having the descriptor “baby-led weaning” in the heading, abstract, or keywords, with no restriction regarding year of publication, location, population, or age group, due to the scarcity of works on the subject. We excluded review articles, editorials, letters to the editor, critical commentaries, and books on the subject, as well as duplicates and articles not available in full, even with the aid of the *Portal de Periódicos* from the Coordination for the Improvement of Higher Education Personnel (CAPES – *Coordenação de Aperfeiçoamento de Pessoal de Nível Superior*).

We selected articles that met the criteria established, analyzed the articles at first by assessing the headings, then read the abstracts and, subsequently, the full article. In the study selection and categorization steps, we developed a cataloging matrix, in which we organized the data for each study. For the analysis and interpretation of results, we read the full texts and elaborated a summary matrix for a qualitative evaluation of information, consisting of complete reference, objective of the study, intervention studied, intervention approach, and model.

## RESULTS

**Figure 1:**
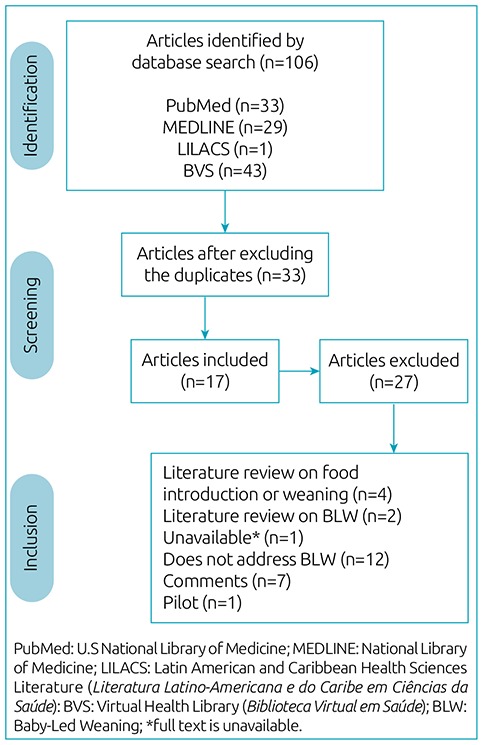
Steps for inclusion and exclusion of articles.

We selected 17 potentially relevant articles that addressed BLW.^14-30^ Figure 1 details the steps adopted to search and choose them. We excluded 27 studies due to incompatibility with the established criteria.

Regarding the design, 6 studies were longitudinal, and 11 were cross-sectional, all in the English language, and published between 2011 and 2017. These articles assessed several topics, revealing the risks and benefits of the approach. The most cited topics were: choking, self-regulation, intake of micro- and macronutrients, start of complementary feeding, weight gain, and family behavior. Tables 1 and 2 summarize general aspects of the cross-sectional and longitudinal studies, respectively, including their authors, year of publication, type of research, and brief description of results.

**Table 1:** Cross-sectional studies selected on the Baby-Led Weaning method.

Authors	Design (n)	Summary of the studies
Brown and Lee^14^	Cross-sectional (655 mothers)	Higher level of maternal schooling; longer breastfeeding; less anxiety; offers fresher and home-prepared foods; respects the satiety.
Brown^16^	Cross-sectional (604 mothers)	Less food restriction, less anxiety; more confidence, and less compulsion.
Cameron et al.^18^	Cross-sectional (199 mothers)	At six months of age, the infants eat with the family, sharing the same foods from the beginning; low iron intake.
Moore et al.^20^	Cross-sectional (n=3,607)	Low understanding of weaning guidelines and low maternal age were responsible for early weaning. Following the BLW approach had a positive influence.
Morison et al.^21^	Cross-sectional (51 children)	Energy intake and the offering of foods with a risk of asphyxia were similar between the BLW and traditional groups. BLW infants eat with family more often, present a higher intake of total and saturated fat, and lower of iron, zinc, and vitamin B12.
Rowan and Harris^22^	Cross-sectional (10 mothers)	There was no change in the dietary patterns of parents, and 57% of the foods offered to the child were the same as those consumed by the family.
Townsend and Pitchford^24^	Cross-sectional (155 parents)	The traditional group showed a preference for sweet foods and the BLW for carbohydrate-rich cereals. Healthier food choices and self-regulation of appetite lead to lower BMI. Incidence of low weight in the BLW group and obesity in the traditional group.
Cameron et al.^26^	Cross-sectional (31 health professionals and 20 mothers)	Health professionals are reluctant to recommend the method. In contrast, mothers are positive in their reports about BLW.
Brown and Lee^27^	Cross-sectional (36 mothers)	The sample clearly showed a positive experience after following the BLW method until the second year.
Brown^28^	Cross-sectional (1,151 mothers)	The risk of choking was the same in the BLW and traditional groups. The higher choking frequency is related to eating less with the hands.
Brown and Lee^29^	Cross-sectional (702 mothers)	Mothers show lower restriction levels when offering to the child the same foods consumed by the family, less pressure and monitoring during mealtime, and less concern with weight.

BLW: baby-led weaning.; BMI: body mass index.

**Table 2:** Longitudinal studies selected on the Baby-Led Weaning method.

Authors	Design (n)	Summary of the studies
Brown and Lee^15^	Longitudinal (298 mothers)	Greater regulation of satiety levels and lower probability of overweight.
Cameron et al.^17^	Longitudinal (23 families)	The BLISS group offered foods with higher iron content and lower choking risk.
Fangupo et al.^19^	Longitudinal (206 children)	There was no significant difference in the number of cases of asphyxia and choking in the BLISS and traditional groups.
Taylor et al.^23^	Longitudinal (166 mothers)	There was no difference regarding BMI between the BLISS and traditional groups. The BLISS group showed less food fussiness and more enjoyment of food.
Wright et al.^25^	Longitudinal (923 children)	Self-feeding is feasible for most babies, but some will develop this skill only at eight months of age. For them, the likelihood of walking without help at one year of age and saying small words is lower.
Arden and Abbott^30^	Longitudinal (15 mothers)	Many parents adopt a mixed method due to insecurity but declare that they follow BLW. Part of a parenting philosophy or used when the traditional approach failed; parents adhere to the method based on its “freedom.”

BLISS: Baby-Led Introduction to SolidS; BMI: body mass index.

## DISCUSSION

### Self-regulation

According to Brown and Lee,^14^ BLW infants were significantly more sensitive to satiety and autonomy (p<0.01), when compared to babies fed in the traditional way. These authors, in another cross-sectional study, with a sample of 702 mother-infant dyads, identified that the mothers who followed the BLW model did not pressure their children as much during meal times, were less concerned with weight, did not directly interfere in the amount of food consumed, and consequently promoted the self-regulation of appetite and satiety.^29^ The authors suggest the BLW method as the standard for complementary feeding since the self-knowledge of satiety and appetite contribute to a healthy dietary and behavioral pattern in the future.

### Intake of macro- and micronutrients

Four studies addressed the intake of macro- and micronutrients in BLW, considering the concerns regarding the adequate consumption of iron and energy. Cameron et al.^18^ highlighted a deficit in iron intake among infants strictly following BLW at the beginning of food introduction, when compared to children whose parents did not solely adhere to the method or who adopted the traditional feeding. According to the authors, parents who practice BLW prioritize breastfeeding up to six months, waiting for the baby to be ready to start feeding; however, they put the consumption of iron-rich foods at risk.

We found similar findings regarding the delay in providing iron-rich foods in the study by Morison et al.,^21^ in which parents who adopted the BLW method offered these foods to their children only five weeks after the traditional group. These authors also assessed energy intake and found that both groups (BLW and traditional feeding) presented similar values but from different sources, underlining a greater energy intake from total and saturated fat in the BLW group.

The intake of zinc, iron, vitamin B12, vitamin C, fiber, and calcium was also lower in this group, although the differences were not significant. According to the authors, the iron intake could be compromised by the emphasis on breastfeeding, as the iron composition in breast milk decreases after the sixth month when compared to fortified formulas. The consumption of vitamin C and calcium was lower than that observed in the traditional group, but above the recommended. As to fiber, there are no recommendations for infants. Morison et al.^21^ also emphasize the high fat intake that results from eating together, risking the consumption of foods not nutritionally appropriate for the age group. Nonetheless, these authors reinforce the benefits of sharing family meals when the foods are adequate in terms of nutrient quality.

Townsend and Pitchford^24^ identified a significant increase in carbohydrate intake among BLW infants when compared to the spoon-fed group. Babies following this approach had a higher preference for cereals from the bottom of the food pyramid, probably because they were easier to chew. In contrast, the traditional group showed a preference for sweets, suggesting that the BLW method promotes healthy eating.

Wright et al.^25^ noted that BLW children only sought foods to self-feed at eight months of age, which creates a nutritional concern, suggesting a less rigid approach in the first few weeks.

### Start of complementary feeding

Moore et al.^20^ conducted a study with 3,607 participants who adhered to the BLW method and found that 50% of the mothers had started complementary feeding before 23 weeks and 50% after 24 weeks. The beginning of complementary feeding at the proper time was related to greater knowledge of guidelines for the baby-led approach (p<0.001) and not with the level of maternal schooling, as usual. The significant determining factors for food introduction and continuation of breastfeeding in this study were: signs of infant development; reaching the recommended age; advice from friends and family; influence of visits from health agents; and babies who often woke up at night. This study also highlighted the popularity of BLW in the United Kingdom and the trust that parents have in the information found on the Internet.

### Weight gain

Several authors associate the BLW approach to weight gain in the baby. Brown and Lee^15^ carried out a study with 298 mother-infant dyads, evaluating the birth weight, weight at six months, and current weight in children whose mean age was 8.34 months and identified that spoon-fed infants were significantly heavier than those in the BLW group (p=0.005). This relationship did not depend on birth weight, duration of breastfeeding, age at the introduction to solid foods, and maternal control. The BLW group comprised 86.5% of normal-weight, 8.1% of overweight, and 5.4% of underweight children. In contrast, the traditional feeding group had 78.3% of normal-weight, 19.2% of overweight, and 2.5% of under-weight children, i.e., this group had a higher percentage of overweight infants. The birth weight, weight at six-month, and current weight were not significantly related to the child’s satiety or the ability for self-regulation. Nevertheless, the current weight of the infant had a significant and inverse association with fussy eating (Pearson: r=-0.171; p=0.003).

Corroborating these findings, Townsend and Pitchford^24^ assessed the body mass index (BMI) of 155 children who followed BLW and traditional feeding. The average percentile in the BLW group was close to the expected (50^th^ percentile), according to the NHS (growth curves in the United Kingdom) (p<0.008) and CDC (growth curves in the United States) (p<0.005) classification systems. On the other hand, the average percentile in the spoon-fed group was considered overweight. Thus, the incidence of obese children was higher in the spoon-fed group compared to the BLW group. The BLW group also had a significant and higher prevalence of underweight children compared to the spoon-fed group (Fisher’s exact test: p=0.02). Therefore, these authors defend the idea that BLW promotes healthier food choices during childhood, protecting children against obesity.

In a study with 702 mothers, Brown and Lee^29^ identified that the increase in child weight was positively correlated with food restrictions, monitoring, pressure during mealtime, maternal control, and concern with the baby’s weight (Pearson: r=0.094; p<0.05). Furthermore, mothers with high food restriction (Pearson: r=0.068; p<0.05) and eating disorders (Pearson: r=0.0103; p<0.01) reported higher levels of concern regarding weight, restriction, and monitoring and realized that their children were bigger (Pearson: r=0.076; p<0.05).

In contrast to these findings, Taylor et al.^23^ conducted a randomized clinical trial with 166 children and found no statistical differences between BMI and energy self-regulation in children who follow BLW, when compared to those from the traditional feeding group. Thus, further prospective controlled studies are necessary to confirm if the BLW method interferes in weight gain.

### Choking

Choking is one of the topics that creates more uncertainty among families and health professionals when children are being fed through the BLW model.^28^ Brown et al.^28^ found no significant associations between the feeding method, the frequency of spoon use (for those following an adapted model), and asphyxia in a study carried out with 1,151 children. However, infants whose parents reported choking and adhered to a traditional approach experienced a significantly higher number of episodes with finger foods (p=0.014) and purees (p=0.002) than those from the BLW group.

Cameron et al.^17^ carried out a comparison between two groups: an exclusive BLW group and another that followed the Baby-Led Introduction to SolidS (BLISS), considered an adaptation of BLW in the face of the concerns regarding the intake of micro- and macronutrients and choking. The authors did not identify statistical differences; however, the BLISS group was less likely to offer foods with a high choking risk (3.24 versus 0.17 serves/day; p=0.027). These data corroborate the findings by Morison et al.^21^ and Fangupo et al.^19^ The latter adds that infants who follow the BLISS approach choked more often at 6 months of age [relative risk (RR) 1.56; 95% confidence interval (95%CI) 1.13–2.17], but with less frequency at 8 months (RR=0.60; 95%CI 0.42–0.87), when compared to the control group – babies on traditional feeding.

Despite the need for more studies, these findings put the practice of BLW at risk, as it exposes the child to potential choking and suffocation.

### Duration of breastfeeding

Few articles discuss the relationship between BLW and the duration of breastfeeding. Moore et al.^20^ performed a survey with 3,607 participants and verified that knowledge about BLW guidelines was associated with exclusive breastfeeding for a longer period (regardless of demographic factors; p<0.001); nonetheless, 80% of mothers stopped exclusive breastfeeding before the baby reached 24 weeks of life and 65% before 17 weeks despite being aware of the guidelines. Young maternal age was a significant factor for early weaning (p=0.014).

These data reinforce the findings by Brown et al.,^14^ which indicate a significantly longer duration of exclusive breastfeeding among mothers who adopted BLW practices compared to those who adhered to the traditional method (p<0.001; 127.36 days for BLW versus 82.11 weeks for the traditional method). Despite the need for more extensive studies, these data suggest the potential longer duration of breastfeeding among those who follow the BLW model.

BLW positively favors family-shared meals, the baby’s satiety, and maternal control regarding the anxiety about the amount of food consumed; promotes more exposure to a greater variety of foods; creates a higher interaction with the food, allowing the exploration of different textures; and starts food introduction at the appropriate age, as suggested by the Brazilian Society of Pediatrics, WHO, and the Dietary Guidelines for children under two years, from the Ministry of Health.

Risks, such as choking and insufficient iron and energy intake, still deserve attention and need to be investigated with further longitudinal randomized controlled studies to ensure the safety of the method when practiced exclusively, bearing in mind that the vast majority of researches obtained their results from questionnaires, without biochemical examinations and anthropometric assessments, which could suggest flaws and biased results.

Considering these findings, and in accordance with the evaluation from the Brazilian Society of Pediatrics, BLW cannot be recommended as the sole method of complementary feeding. The combination of both methods, BLW and traditional, could lead to a feeding model that enables the infant’s autonomy, with more active participation during meals, but guided and assisted by parents, which would be a safe alternative for the integral development of the baby.
